# Proteins involved in difference of sorbitol fermentation rates of the toxigenic and nontoxigenic *Vibrio cholerae *El Tor strains revealed by comparative proteome analysis

**DOI:** 10.1186/1471-2180-9-135

**Published:** 2009-07-09

**Authors:** Ruibai Wang, Hongzhi Zhang, Haiyan Qiu, Shouyi Gao, Biao Kan

**Affiliations:** 1 State Key Laboratory for Infectious Disease Prevention and Control, Department of Diarrheal Diseases, National Institute for Communicable Disease Control and Prevention, Chinese Center for Disease Control and Prevention, Beijing 102206, PR China

## Abstract

**Background:**

The nontoxigenic *V. cholerae *El Tor strains ferment sorbitol faster than the toxigenic strains, hence fast-fermenting and slow-fermenting strains are defined by sorbitol fermentation test. This test has been used for more than 40 years in cholera surveillance and strain analysis in China. Understanding of the mechanisms of sorbitol metabolism of the toxigenic and nontoxigenic strains may help to explore the genome and metabolism divergence in these strains. Here we used comparative proteomic analysis to find the proteins which may be involved in such metabolic difference.

**Results:**

We found the production of formate and lactic acid in the sorbitol fermentation medium of the nontoxigenic strain was earlier than of the toxigenic strain. We compared the protein expression profiles of the toxigenic strain N16961 and nontoxigenic strain JS32 cultured in sorbitol fermentation medium, by using fructose fermentation medium as the control. Seventy-three differential protein spots were found and further identified by MALDI-MS. The difference of product of fructose-specific IIA/FPR component gene and mannitol-1-P dehydrogenase, may be involved in the difference of sorbitol transportation and dehydrogenation in the sorbitol fast- and slow-fermenting strains. The difference of the relative transcription levels of pyruvate formate-lyase to pyruvate dehydrogenase between the toxigenic and nontoxigenic strains may be also responsible for the time and ability difference of formate production between these strains.

**Conclusion:**

Multiple factors involved in different metabolism steps may affect the sorbitol fermentation in the toxigenic and nontoxigenic strains of *V. cholerae *El Tor.

## Background

*Vibrio cholerae *is the causative agent of the diarrheal disease cholera. Out of the 200 serogroups of *V. cholerae*, only two biotypes of serogroup O1 (classical and El Tor) and serogroup O139 cause severe diarrhea and epidemic cholera [1], although not all strains in these two serogroups are pathogenic. Toxigenic and nontoxigenic *V. cholerae *strains are genetically diverse. The toxigenic strains form a genetically homogenous group, while nontoxigenic strains are heterogeneous and may have diverse origins [2-4]. The nontoxigenic strains, which are usually isolated from environmental sources such as sewage, oysters, and brackish water, do not carry cholera toxin (CT) and other major virulence genes necessary for human pathogenesis [5].

*V. cholerae *is capable of metabolizing many types of carbohydrates. Previously, we found that not only is D-sorbitol metabolized by *V. cholerae*, but it is also fermented at different rates by the toxigenic and nontoxigenic El Tor strains. The toxigenic strains have a low sorbitol fermentation rate and are called slow-fermenting strains, whereas the nontoxigenic strains have a faster sorbitol fermentation rate and are called fast-fermenting strains [6]. The sorbitol fermentation test is included in the Phage-biotyping scheme, which consists of phage typing and biochemical typing and is developed in 1970s in China. This scheme is used to distinguish and type the El Tor strains which are pathogenic and are potential to cause epidemic or not [6]. It is found that the O1 El Tor strains isolated from patients and environmental samples in epidemics are toxigenic and sorbitol slow-fermenting, whereas the strains isolated from environment in non-epidemic periods are nontoxigenic and fast-fermenting.

In some bacteria, D-sorbitol is transported into the cell via the sorbitol specific phosphotransferase system (PTS) or some non-sorbitol specific PTS, and then it is transformed from sorbitol-6-phosphate to fructose-6-phosphate and enters the fructose/mannitol metabolism pathway. All genes involved in the fructose/mannitol metabolism pathway in *V. cholerae *have been identified and annotated on the genome [7], but the genes involved in sorbitol transportation and transformation are unknown http://www.genome.jp/dbget-bin/show_pathway?vch00051, though a previous study identified the differential proteins expressed in the presence or absence of sorbitol, based on which only the sorbitol induced proteins could be found [8].

An investigation into the mechanism behind the different fermentation rates in toxigenic versus nontoxigenic *V. cholerae *strains may help to further the understanding of their genetic and evolutionary differences. Here, we used nuclear magnetic resonance (NMR) and two-dimensional gel electrophoresis (2-DE) to identify differences in metabolites and proteins involved in sorbitol fermentation between toxigenic (sorbitol slow-fermenting) and nontoxigenic (sorbitol fast-fermenting) *V. cholerae *El Tor strains. Proteomics is a useful high-throughout technique and has been used in *V. cholerae *to construct proteome reference map [9], protein expression analysis in the different culture environments [8,10,11] and in the human host environment [12]. Large genetic differences exist between the toxigenic and nontoxigenic *V. cholerae *based on the comparative genomic hybridization [13], accordingly protein components of these strains will be much more divergent. The direct comparison of protein profiles of the fast- and slow-fermenting strains cultured in sorbitol fermentation medium will lead the confusion and misunderstanding of the proteins associated with the mechanisms of fermentation difference. Fructose and sorbitol metabolisms share the same pathway after the fructose-6-phosphate step, and we found no differences in fructose fermentation rates between the sorbitol fast- and slow-fermenting strains, therefore in this study we used fructose as a control when comparing protein profiles, to exclude proteins constitutively involved in sugar metabolism. This approach allowed to identify differences in protein expression associated with sorbitol metabolism difference in the toxigenic and nontoxigenic *V. cholerae *strains. Differences of formate production, fructose-6-phosphate production and subsequent metabolism were found to be causative mechanisms in the sorbitol fermentation difference in the toxigenic and nontoxigenic *V. cholerae *strains.

## Methods

### Bacterial Strains

Two *V. cholerae *strains of serogroup O1 El Tor (N16961 and JS32) were used to compare protein expression profiles by 2-DE analysis. N16961 is a toxigenic strain whose complete genome was previously published [7], while JS32 is a nontoxigenic strain isolated in 1982 in China. An additional eight toxigenic strains (97005, LN2001-5, GD97-73, ZJ62-10, D118, 93–284, WUJIANG-2 and 63–12) and three nontoxigenic strains (V05-18, 79327 and 60–61) isolated in China were also included in this study (Table 1).

**Table 1:** The strains used in this study and their characters of major virulent genes

Strains	*ctxAB*	*tcpA*	*hlyA*	Year of isolation	Location
60–61	-	+	+	1977	Zhejiang
79327	-	-	+	1979	Hebei
JS32	-	-	+	1982	Jiangsu
V05-18	-	+	+	2005	Guangdong
D118	+	+	+	1961	Guangdong
Dec-63	+	+	+	1961	Yunnan
ZJ62-10	+	+	+	1962	Zhejiang
N16961	+	+	+	1971	Bangladesh
WUJIANG-2	+	+	+	1980	Jiangsu
93–284	+	+	+	1993	Guangdong
GD97-73	+	+	+	1997	Guangdong
97005	+	+	+	1997	Hebei
LN2001-5	+	+	+	2001	Liaoning

### Sorbitol and fructose fermentation tests

Fresh colonies cultured on Luria-Bertani (LB) agar were selected and inoculated statically in 1 ml LB broth at 37°C for 2 hours, to reach the OD_600 _of 0.5 or 1 × 10^7^CFU/ml equivalently. Then 100 μl cultures were transferred into 3 ml fermentation media (0.01% peptone, 5% NaCl, 2% sorbitol or fructose, and 0.025% phenol red; pH 8–9) and inoculated statically at 37°C. Sugar fermentation was measured as the color change in the medium 4 and 8 hours post-inoculation (yellow, fast fermentation or a positive test; red, slow fermentation or a negative test) [6]. Considering the high concentration of sorbitol in the fermentation medium, fructose at a similar concentration was used as a control sugar in the proteome analysis to eliminate differences in nutrient usage, osmotic pressure and pH in the media with and without sorbitol. pH of the fermentation medium was measured with CPpH 59003-05 (Cole).

### ^1^H-NMR

One milliliter of the fermentation media cultured with the test strains was collected and centrifuged at 10,000 × *g *at room temperature for 10 min to clarify the supernatant. The^1^H resonance of D_2_O (10%) was used to lock the field and for shimming. Tetramethylsilane was used as internal standard. NMR spectra were recorded on a Varian INOVA 600 spectrometer (Varian Inc, USA) operating at 60 MHz with the following parameters: pulse 55.1 degrees, mixing 0.15 sec, acquire time 4.573 sec, 7 kHz spectral width, line broadening 0.5 Hz, 128 repetitions, FT size 131072.

### Comparative proteome analysis

*V. cholerae *strains N16961 and JS32 were cultured in 400 ml sorbitol or fructose fermentation media. The *V. cholerae *cell precipitates were washed with precooled low salt PBS (3 mM KCl, 1.5 mM KH_2_PO_4_, 68 mM NaCl, 9 mM NaH_2_PO_4_) and disrupted and solubilized using lysis solution (7 M Urea, 2 M Thiourea, 4% CHAPS, 50 mM DTT) and sonicated for 2 min on ice using the Sonifier 750 (S&M0202, Branson Ultrasonics Corp., Danbury, CT, USA). After centrifuging at 100,000 × *g *15°C for 45 min, supernatant aliquots were stored at -70°C and the protein concentration was determined with the PlusOne 2-D Quant Kit (Amersham Pharmacia, Sweden).

2-DE was performed using the Immobiline/polyacrylamide system and 18 cm IPG strips (pH ranges 4 to 7) (Amersham Pharmacia Biotech, Sweden). Seven hundred microgram samples were loaded, and isoelectric focusing was conducted at 20°C for 58,000 Vhrs (maximum voltage of 8,000 V) on IPGphor (Amersham Pharmacia Biotech, Sweden). For the second dimension, vertical slab SDS-PAGE (12.5%) was used (Bio-Rad protean II Xi, Bio-Rad laboratories, USA). Gels were stained using Colloidal Coomassie Blue G-2500 (5 g G-250, 170 ml methanol, 212.5 ml 40% ammonium sulfate, 15 ml phosphoric acid, and 102.5 ml purified water). Three sample preparations were made for every strain, and each sample was repeated at least twice. Images were analyzed using the Image-Master 2D Elite (Amersham Pharmacia Biotech, Sweden).

### In-gel protein digestion, MALDI-TOF-MS and protein identification

Protein spots of interest were excised from the gels. After destaining, gel pieces were digested with trypsin (Roche, Germany) for 12 h at 37°C. The extracts were dried and resolubilized in 2 μl of 0.5% TFA. Peptide mass fingerprinting (PMF) measurements were performed on a Bruker Reflex™ III MALDI-TOF mass spectrometer (Bruker Daltonik GmbH, Bremen, Germany) working in reflectron mode with 20 kV of accelerating voltage and 23 kV of reflecting voltage. A saturated solution of α-Cyano-4-hydroxycinnamic acid (CHCA) in 50% acetonitrile and 0.1% trifluoroacetic acid (TFA) was used for the matrix. Mass accuracy for PMF analysis was 0.1–0.2 Da with external calibration; internal calibration was carried out using enzyme autolysis peaks, and the resolution was 12,000. Database searches were performed using the software Mascot v1.7.02 (Matrix Science Ltd.) licensed in-house http://mascot.proteomics.com.cn/search_form_PMF.html against the database of *V. cholerae *N16961 (Version Vib CLEAN 040921, 3814 sequences). Monoisotopic peptide masses were used to search the databases with a mass tolerance of 100 ppm and one partial cleavage. Oxidation of methionine and carbamidomethyl modification of cysteine was considered. Scores greater than 48 were significant (p < 0.05), with more than five peptides matched and sequence coverage greater than 15%.

### Sequencing of the gene VCA0518

The gene VCA0518 (designated in the genome of N16961, GenBank Accession Number NC002506), which corresponds to the fructose-specific IIA/FPR component (PTS system, FIIA), was amplified from all tested strains using primers 5' GCG CTG GAT TTA AGG TGA TGG 3' and 5' TCG CCT ATA GAG GCA GAC AGG 3' and sequenced. The sequences were searched in the CDD database (V2.16-27036PSSMs, http://www.ncbi.nlm.nih.gov/Structure/cdd/wrpsb.cgi) for conserved domain analysis.

### Quantitative real-time PCR (qRT-PCR)

Total RNA from N16961 and JS32 cultured in sorbitol fermentation media was extracted at the inoculation time points 2, 4, 6 and 8 h with the RNeasy Mini Kit (QIAGEN). The following primer pairs were used in reverse transcription and amplification of genes: 5' CCG CAG GAA TCG TGT TGA TGT AG 3' and 5' GAA TCC GTT GTC CGT GAA GAA GG 3' for pyruvate dehydrogenase subunit E1 (VC2414); 5' CAC GAC GCT GGC TAC ATC AAC 3' and 5' ACC ATA CGG ATA CCA CCA TTA GGG 3' for pyruvate formate-lyase 1 activating enzyme (PFL) (VC1866); and 5' AAG ATT GGT GTG ATG TTT GGT A 3' and 5' CAC TTC TTC GCC TTC TTT GA 3' for the internal standard *recA *gene. The reaction was performed using the SYBR premix Ex Taq™ (TaKaRa, Dalian, China). The 2^-ΔΔCt ^method was used to calculate relative expression of the VC18166 gene to the VC2414 gene in the N16961 and JS32 strains, and normalized with the control gene *recA*. ΔΔCt = (Ct_VC1866 _- Ct_VC1866recA_) - (Ct_VC2414 _- Ct_VC2414recA_). Ct_VC1866recA _and Ct_VC2414recA _indicating the Ct values of *recA *simultaneously amplified with VC1866 and VC2414, Ct_VC1866 _and Ct_VC2414 _indicate the Ct values of VC1866 and VC2414.

## Results

### Dynamic change of the fermentation medium pH

**Figure 1:**
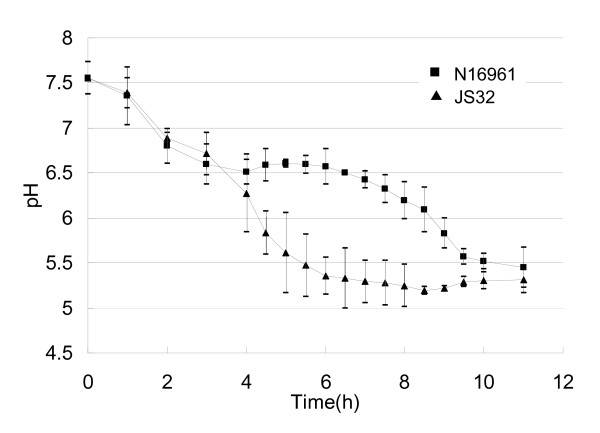
**pH-time curves of toxigenic strain N16961 and nontoxigenic strain JS32 on sorbitol fermentation media**.

We measured the pH of the sorbitol fermentation media of the strains during the fermentation test, by extracting 5 ml of the media serially at each time point, from a volume of 400 ml culture of each strain. The pH-time curves (Fig. 1) demonstrate that the JS32 sorbitol fermentation medium pH dropped gradually over time, while that of N16961 leveled off at pH 6.5 for about 2 hours before dropping again. The change in pH was consistent with the sorbitol fermentation test, showing that nontoxigenic strains display positive results earlier than toxigenic strains [6].

### ^1^H-NMR analysis

**Figure 2:**
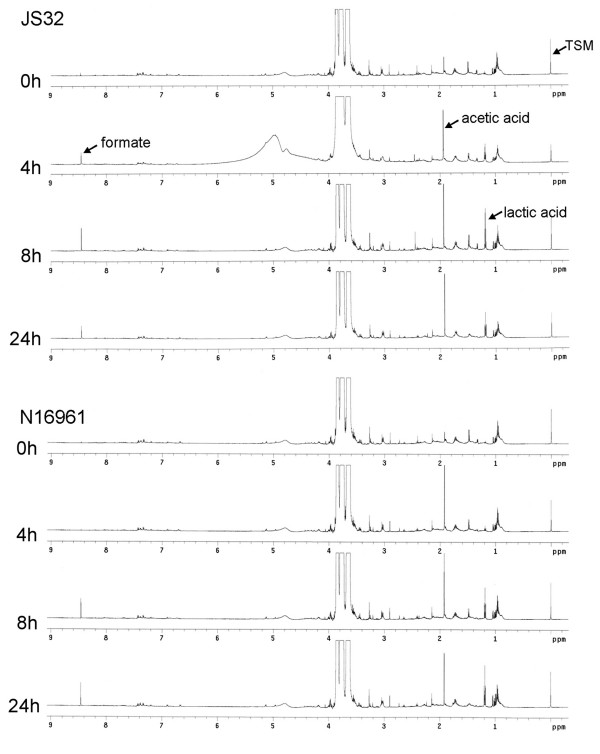
**^1^H-NMR spectra of JS32 and N16961 sorbitol fermentation medium**. Samples were collected at four time points: the starting time (0 h), the JS32 color change (4 h), the N16961 color change (8 h), and 24 hours. Formate could be seen at 4 h in JS32, while there was no formate peak in N16961.

In order to understand the differences in pH observed for the toxigenic and nontoxigenic strains, we examined changes in medium components using ^1^H-NMR. The majority of the components in the sorbitol fermentation media exhibited similar depletion or formation for JS32 and N16961 (Fig. 2). One exception was the appearance of two volatile compounds (formate and lactic acid). Formate appeared in the JS32 culture earlier than in the N16961 culture, and the different production rates of formate between these two *V. cholerae *strains were consistent with their pH changes and fermentation rates. At the time of color change in the JS32 fermentation sample, the concentrations of acetic acid and formate in the medium were 30.53 mg/L and 16.86 mg/L (0.509 mmol/L and 0.367 mmol/L, respectively). In contrast, the acetic acid concentration in N16961 fermentation media was 24.37 mg/L (0.406 mmol/L), and formate was below the level of detection.

### Comparative proteomic analysis

At the positive time point of the sorbitol fermentation test of JS32 (4 hours), whole cell proteins from four different cultures were prepared and separated by 2-DE. These protein profiles were designated FN, SN, FJ and SJ, indicating samples prepared from N16961 in fructose, N16961 in sorbitol, JS32 in fructose, and JS32 in sorbitol fermentation medium, respectively.

**Figure 3:**
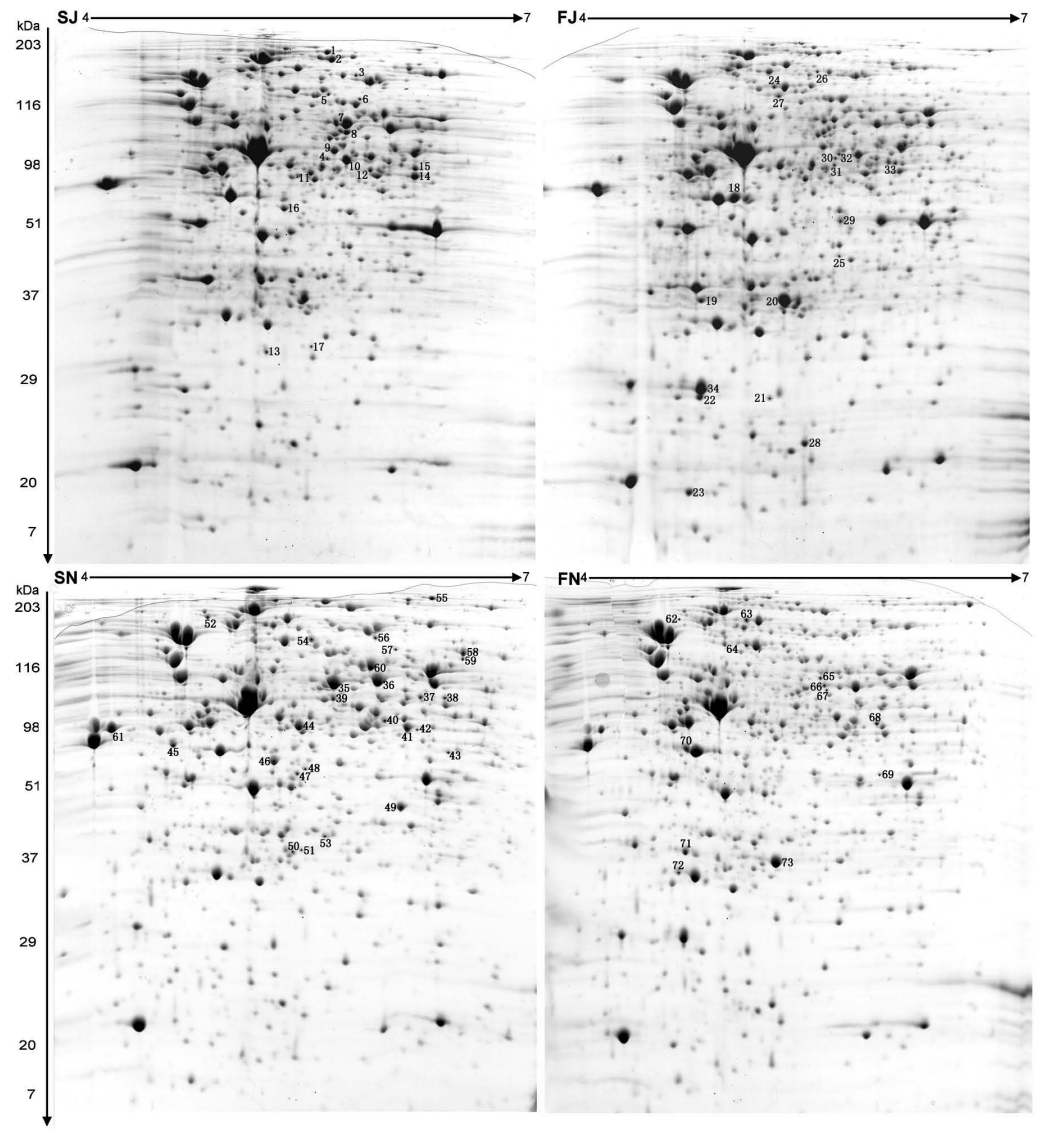
**2-DE gels of whole cell proteins of *V. cholerae *strains JS32 and N16961 cultured in sorbitol and fructose fermentation media**. The comparative proteins (comparison between SN/FN and SJ/FJ) were marked and numbered on their more abundant maps.

On the SN and FN proteome profiles, 901 and 903 spots were identified, respectively, but only 39 spots had changed in abundance, in them 27 were more abundant in N16961 cultured in sorbitol fermentation medium (SN), 12 were more abundant in sample of FN (Fig. 3) [also see Additional file 1]. Such similarity also existed in the SJ and FJ profiles, with 34 differential spots found, 17 were more abundant in samples of SJ and FJ respectively (Fig. 3) [also see Additional file 1]. All of the 73 differential protein spots were analyzed by MALDI-MS, and 71 spots significantly matched known proteins (one spot of FJ and one spot of SN were not identified) [see Additional file 1]. Sixty-two percent of the spots were identified as proteins involved in energy metabolism and central intermediary metabolism, and six spots were identified as transport/binding proteins.

Out of 73 total differential spots identified in the SN/FN and SJ/FJ comparisons, 10 common signified potential proteins of these two comparison groups may be involved in the difference between the sorbitol and fructose metabolism pathway: amino acid ABC transporter, perosamine synthase, malate dehydrogenase, aminotransferase NifS, heat shock protein HtpG, succinyl-CoA synthase, FIIA, glycerol kinase, pyruvate dehydrogenase, and oxygen-insensitive NAD(P)H nitroreductase. Three of these proteins (glycerol kinase, oxygen-insensitive NAD(P)H nitroreductase, and FIIA) were more abundant in sorbitol medium.

**Figure 4:**
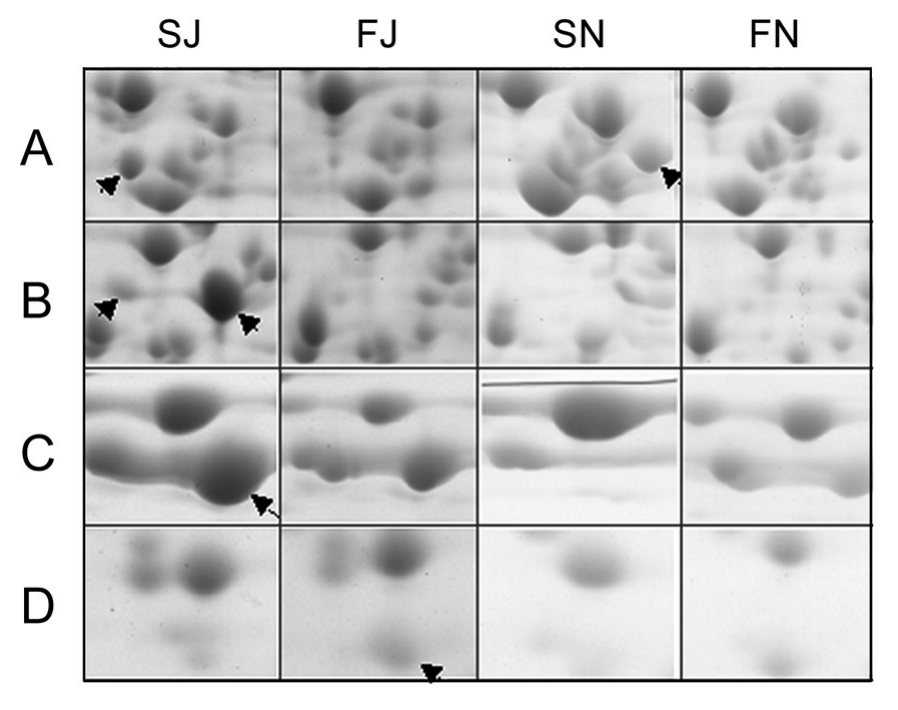
**Part view of four differential protein spots related to sorbitol transportation and acid metabolite production**. The spots corresponding to the proteins are indicated with arrows. A, fructose specific IIA/FPR component; B, mannitol-1-P dehydrogenase; C, pyruvate dehydrogenase; D, pyruvate formate-lyase 1 activating enzyme.

Two proteins within the identified 73 spots, the FIIA protein of PTS system and mannitol-1-P dehydrogenase (MtlD), may be involved in the transportation and transformation of sorbitol. FIIA was a common differential protein observed in the comparisons of SN/FN and SJ/FJ, both of which were more abundant in the sorbitol medium than in the fructose medium (Fig 4A). MtlD was more abundant in sorbitol than in fructose fermentation medium of the sorbitol fast-fermenting strain JS32, but was not found difference in SN/FN comparison of the sorbitol slow-fermenting strain N16961 (Fig. 4B), suggesting its potential role in the sorbitol metabolism difference between these two strains. Two different p*I *forms of it were also found in SJ sample (Fig. 4B). Two proteins, pyruvate dehydrogenase and PFL, were also found differences in the comparison of sorbitol to fructose fermentation in JS32 and N16961, pyruvate dehydrogenase was more abundant in SJ of SJ/FJ comparison but less abundant in SN of SN/FN comparison, and PFL was more abundant in both FJ and FN (Fig. 4C and 4D). These two enzymes were both involved in pyruvate transformation, and PFL catalyzes pyruvate to produce formate. Their different expression may suggest their roles in formate production in the sorbitol fast- and slow-fermenting strains. In addition, the haemolysin and hcp proteins, which are related to *V. cholerae *pathogenicity, were also abundant spots on the SN gel, showing higher expression levels in N16961.

### Sequencing of the VCA0518 gene

**Figure 5:**
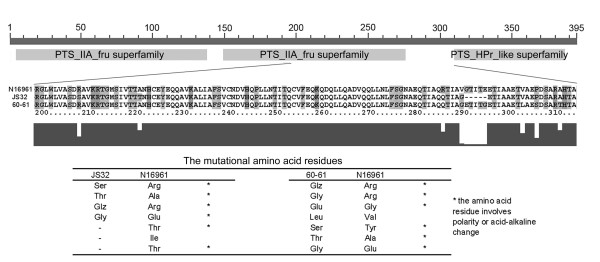
**The conserved domains and homology analysis of VCA0518 encoding product of the toxigenic strain N16961, nontoxigenic strains JS32 and 60–61**. The thick line on the top of the figure means the whole length of the predict peptide chain of the VCA0518 product. The conserved domains are marked with the grey rectangles under the line. Fourteen mutated residues distributed at six sites from amino acids 200 to 310 are shown below the domain map. Residue changes are listed on the bottom of the figure. Amino acid residues with polarity or acid-alkaline changes are marked with *.

Due to the observed differences on the 2-DE gels (the VCA0518 gene product, FIIA component), the VCA0518 gene from all toxigenic and nontoxigenic strains studied were amplified and sequenced (GenBank: EF581766 to EF581778). All of the sequences contained three predicted conserved domains: the fructose specific PTS EIIA component, the EIIA component of PTS, and the HPr protein. The sequences of the nine toxigenic strains were highly similar but differed from the nontoxigenic strains, while three of four nontoxigenic strains had identical sequences. A comparison of amino acid residues of the nontoxigenic and toxigenic strains revealed changes mainly localized at the spacer region between the latter two domains. Nearly all of these residues involved changes in the polarity or acid-alkalinity of the amino acid (Fig. 5). Three of the four nontoxigenic strains (JS32, 79327 and V05-18) lacked a 15 nucleotide (nt) region (AGCTGTGGGAACGAT) from 861 to 875, and the p*I*s of their proteins changed from 5.88 to 5.75. This data was consistent with the appearance of the FIIA protein spots on the 2-DE gels. The nontoxigenic strain 60–61 did not lack the 15 nt fragment, but amino acid mutations placed it in the farthest phylogenetic cluster from the other strains (data not shown).

### qRT-PCR of VC1866 and VC2414

**Figure 6:**
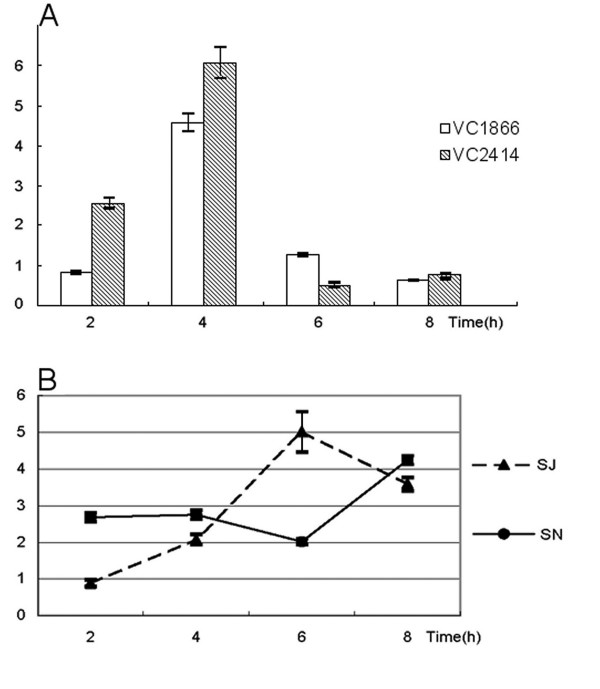
**Transcription level of VC1866 and VC2414 genes tested by qRT-PCR in strains JS32 and N16961 cultured in sorbitol fermentation medium at different time points**. (A) The relative levels of VC1866 and VC2414 in comparison of JS32 to N16961. Both VC1866 and VC2414 were more highly transcripted in JS32 than in N16961 (B) The transcription ratios of VC1866 to VC2414 in JS32 and N16961 respectively.

PFL (VC1866) and pyruvate dehydrogenase (VC2414) were identified as spots in the proteomic analysis (Fig. 4) and are involved in the production of fermentation acids. We monitored transcription levels at four time points during the fermentation assay. The transcription levels of both VC1866 and VC2414 of JS32 were higher than those of N16961 in sorbitol fermentation medium at 4 hrs and reversed at 8 hrs (Fig. 6A). When comparing the relative transcription levels of VC1866 to VC2414 of JS32 and N16961 (Fig. 6B), we found that the relative transcription of VC1866 of JS32 was higher than of N16961 at all time points. JS32 transcription of VC1866 reached a peak five-fold increase at 6 hrs, whereas N16961 transcription was only increased two-fold. No wonder the fast-fermenting strain JS32 showed much higher production of formate than did the slow-fermenting strain N16961.

## Discussion

Nontoxigenic *V. cholerae *strains ferment sorbitol at a faster rate than toxigenic strains, one of phenotyping included in the Phage-biotyping, which has been widely used as a typing scheme in cholera surveillance for many years in China and has been confirmed by thousands of strains [6]. To understand the mechanism of this difference in sorbitol fermentation rate, here we compared the expression of proteins involved in sorbitol fermentation in toxigenic and nontoxigenic strains. The proteome profiles of the cells cultured in sorbitol and fructose medium were very similar with few differential spots, indicating that the status of the cells in these two conditions was similar. Therefore, we could subtract the most commonly expressed constitutive proteins not related to sorbitol fermentation when comparing SN/FN and SJ/FJ. This approach identified two PTS proteins and two proteins involved in formate production.

In general, the specificity of sugar PTSs lies in their EIIA component, while the HPr protein and EI enzyme are encoded by independent genes and are commonly used by different sugar PTS systems. In the conservative domain analysis of the *V. cholerae *VCA0518 gene, we found that this EIIA component was larruping and it contained three conservative domains, two of which are not sugar-specific. The sequences of the three domains were almost completely identical for all tested strains, further demonstrating their highly conserved nature. We conjectured that the low specificity of the co-expressed HPr and EIIA domains endowed the VCA0518 gene product with a role in sorbitol utilization. Contrary to the conservation of the domains, the entire VCA0518 gene sequences of the 13 tested strains showed obvious differences between the toxigenic and nontoxigenic strains, with the variable amino acid residues located at the spacer region between the domains. These differences may impact the steric conformation and the regulation of this protein, and further impact the efficiency of sorbitol transportation. The regulation of transcription, which maybe also affects the expression of VCA0518 in the sorbitol fast-fermenting and slow-fermenting strains, should also be considered

MtlD catalyses the transformation of mannitol-1-P to fructose-6-P, the later enters the fructose metabolism pathway. Mannitol and sorbitol are very similar in molecular structure. In *Pseudomonas fluorescens*, sorbitol is transported by the mannitol PTS system and transformed by polyol dehydrogenase, which has a broad substrate spectrum [14,15]. In a previous study we confirmed the transcriptions of the N16961 VCA1046 gene in sorbitol and mannitol fermentation media [16]. Here, our results indicate that two non-sorbitol specific PTSs are involved in the *V. cholerae *sorbitol utilization process. This may be similar to the uptake of L-sorbose in *Lactobacillus casei *where L-sorbose is mainly taken up via EII^Sor ^and EII^Man ^plays a secondary role [17]. In *Bacillus subtilis*, MtlD is required for sorbitol assimilation in addition to the *gut *operon [18]. Interestingly, both of these PTSs are located on chromosome II of *V. cholerae*. Several studies indicate that the two chromosomes of *V. cholerae *are heterologous and that chromosome II may be a megaplasmid captured by an ancestral *V. cholerae *[7]. The ability to ferment sorbitol used to differentiate *V. cholerae *strains may provide clues as to both the origins and genetic variation of the toxigenic and nontoxigenic strains.

The traditional sorbitol fermentation test is a phenotypic method using phenol red as the indicator. In our study, we showed that the observed differences in sorbitol fermentation rates were the result of changes in the production rate of formate in the fast-fermenting and slow-fermenting strains. The fact that the ratio of formate to acetic acid was not consistent between the two strains also indicated that, besides the differences early in the metabolic pathway (including the transportation and transformation of sorbitol), pyruvate catabolism could be different in sorbitol fermentation in the toxigenic and nontoxigenic strains. Both pyruvate dehydrogenase and PFL can catalyze the transformation of pyruvate to acetyl-CoA, but they have different electron acceptors and outputs. Their activities affect the relative proportion of the end products [19]. Pyruvate dehydrogenase produces CO_2 _in addition to acetyl-CoA, while formate is the product of PFL. In the proteomic and qRT-PCR analyses of this study, the respective expression and transcription levels of these two genes were significantly different in the fast-fermenting JS32 and slow-fermenting N16961. Consistent with this fact was that formate was produced earlier in JS32 than in N16961. In a previous study, we had confirmed that the sequences of VC1844 including the promoter region of the toxigenic and nontoxigenic strains could be identical (data not shown). The differential transcription or metabolism of pyruvate was not at VC1844 gene level and there must be a regulation mechanism, which acts at the pyruvate point, differs between the toxigenic and nontoxigenic strains.

The most important difference between the toxigenic and nontoxigenic strains is the presence or absence of the cholera toxin gene *ctxAB*. When we deleted *ctxAB *from the toxigenic strains or complemented *ctxAB *via plasmid into the nontoxigenic strains, we did not observe the reversion of the sorbitol fermentation rate when comparing the mutants with the wild-type strains (data not shown). In the proteomic analysis, we identified two virulence-related proteins. Among them, hemolysin has a predominant role in lethality and confers *V. cholerae *the ability to prevent clearance and establish prolonged colonization without a requirement for cholera toxin or toxin-coregulated pili [20,21]. *V. cholerae *Hcp protein is a 28-kDa secreted protein regulated coordinately with hemolysin. The expression of both proteins has been shown to promote expression of virulence determinants *in vivo *and increase LD50 in the infant mouse cholera model [22,23]. Consistent with their co-regulation relationship, both hemolysin and hcp were more abundant in the N16961 sorbitol culture profiles, suggesting that sorbitol induction and metabolism may have relationship with the regulation of the expression of virulent elements in *V. cholerae*.

## Conclusion

We carried out a comparative analysis of the differences induced by sorbitol between toxigenic (sorbitol slow fermentation) and nontoxigenic (sorbitol fast fermentation) *V. cholerae *strains. Our results suggest that the differential expression of the FIIA protein and MtlD of mannitol PTS demonstrate changes in the transportation and metabolism of sorbitol, and that pyruvate dehydrogenase and PFL relate to the different production rate of the acid metabolites. The contribution and functional mechanisms of these proteins in the *V. cholerae *sorbitol fermentation pathway in toxigenic and nontoxigenic strains will require further study.

## Authors' contributions

RW carried out the main part of experiments in this study and drafted the manuscript, HZ carried out qRT-PCR and participated in discussion in preparing the manuscript, HQ participated in cultures and sample preparation, SG and BK designed and coordinated the study, and BK revised the manuscript. All authors read and approved the final manuscript.

## Supplementary Material

Additional file 1 **The differential protein spots identified by PMF**. In this table the protein spots with the differential abundance in the proteome comparisons of SJ/FJ and SN/FN in sorbitol and fructose fermentation media respectively, and their PMF identification results, were listed. Click here for file
