# Supplementary material for: Proteins involved in difference of sorbitol fermentation rates of the toxigenic and nontoxigenic Vibrio cholerae El Tor strains revealed by comparative proteome analysis
Source: BMC Microbiol. 2009 Jul 9;9:135. doi: 10.1186/1471-2180-9-135 (PMC2714520; doi:10.1186/1471-2180-9-135)
Supplement: Additional file 1 — The differential protein spots identified by PMF. In this table the protein spots with the differential abundance in the proteome comparisons of SJ/FJ and SN/FN in sorbitol and fructose fermentation media respectively, and their PMF identification results, were listed. [file 1471-2180-9-135-S1.doc]

**Additional file 1**

Table. The differential protein spots in SJ/FJ and SN/FN comparisons identified by PMF

| No. ^#^ | NCBI GI Identifier | Locus tag | Gene symbol | Protein description | Theoretical mass | Theoretical pI | Peptides matched | Peptides unmatched | Sequence coverage (%) | Score* | Biological role |
| --- | --- | --- | --- | --- | --- | --- | --- | --- | --- | --- | --- |
| Protein spots more abundant of JS32 cultured in sorbitol fermentation medium compared to fructose medium (SJ/FJ comparison) | | | | | | | | | | | |
| 1 | gi|9655036 | VC0604 | *acnB* | aconitate hydratase 2 | 93.6 | 5.3 | 39 | 21 | 65 | 248 | Energy metabolism |
| 2 | gi|9656988 | VC2414 | *aceE* | pyruvate dehydrogenase, E1 component | 99 | 5.5 | 37 | 22 | 63 | 296 | Energy metabolism |
| 3 | gi|9654692 | VC0281 | *cadA* | lysine decarboxylase, inducible | 82.9 | 5.4 | 17 | 10 | 63 | 170 | Energy metabolism |
| 4 | gi|9658490 | VCA1046 | *mtlD* | mannitol-1-phosphate 5-dehydrogenase | 42.3 | 5.3 | 14 | 8 | 64 | 156 | Energy metabolism |
| 5 | gi|9654915 | VC0487 | *fbaA* | glucosamine--fructose-6-phosphate aminotransferase | 67.1 | 5.4 | 24 | 15 | 62 | 217 | Central intermediary metabolism |
| 6 | gi|9658523 | VCA1077 |  | conserved hypothetical protein | 67.1 | 5.6 | 18 | 12 | 60 | 181 | Hypothetical proteins |
| 7 | gi|9657495 | VCA0108 |  | conserved hypothetical protein | 55.6 | 5.3 | 18 | 11 | 62 | 124 | Hypothetical proteins |
| 8 | gi|9658166 | VCA0744 | *gplK* | glycerol kinase | 55.7 | 5.4 | 18 | 11 | 62 | 149 | Energy metabolism |
| 9 | gi|9654996 | VC0566 | *htrA* | protease DO | 48.4 | 5.6 | 17 | 16 | 52 | 179 | Protein fate |
| 10 | gi|9658490 | VCA1046 | *mtlD* | mannitol-1-phosphate 5-dehydrogenase | 42.3 | 5.3 | 18 | 17 | 51 | 185 | Energy metabolism |
| 11 | gi|9656633 | VC2085 | *sucC* | succinyl-CoA synthase, beta subunit | 41.4 | 5.2 | 9 | 8 | 53 | 101 | Energy metabolism |
| 12 | gi|9657923 | VCA0518 | *fruB* | PTS system, fructose-specific IIA/FPR component | 42.6 | 6 | 9 | 9 | 50 | 85 | Transport and binding proteins |
| 13 | gi|9656800 | VC2240 |  | decarboxylase | 19.9 | 5 | 6 | 9 | 40 | 76 | Central intermediary metabolism |
| 14 | gi|9654653 | VC0244 | *rfbE* | perosamine synthase | 41.1 | 6 | 15 | 10 | 60 | 151 | Cell envelope |
| 15 | gi|9654652 | VC0243 | *rfbD* | GDP-mannose 4,6-dehydratase | 42.1 | 5.9 | 12 | 25 | 32 | 102 | Cell envelope |
| 16 | gi|9654855 | VC0432 | *mdh* | malate dehydrogenase | 37 | 7.4 | 10 | 4 | 71 | 162 | Energy metabolism |
| 17 | gi|9658265 | VCA0836 |  | hexapeptide-repeat containing-acetyltransferase | 21.2 | 5.9 | 6 | 3 | 67 | 86 | Central intermediary metabolism |
| Protein spots more abundant of JS32 cultured in fructose fermentation medium compared to sorbitol medium (SJ/FJ comparison) | | | | | | | | | | | |
| 18 | gi|9658440 | VCA0998 |  | NADH-dependent flavin oxidoreductase, Oye family | 38.3 | 4.8 | 9 | 11 | 45 | 115 | Central intermediary metabolism |
| 19 | gi|9654401 | VC0010 |  | amino acid ABC transporter, periplasmic amino acid-binding portion | 27.3 | 5 | 6 | 17 | 26 | 69 | Transport and binding proteins |
| 20 | gi|9658051 | VCA0637 |  | oxygen-insensitive NAD(P)H nitroreductase | 24.1 | 5.1 | 9 | 11 | 45 | 111 | Central intermediary metabolism |
| 21 | gi|9656660 | VC2111 |  | conserved hypothetical protein | 18.4 | 4.9 | 6 | 5 | 55 | 105 | Hypothetical proteins |
| 22 | gi|9656377 | VC1849 | *ppiB* | peptidyl-prolyl cis-trans isomerase B | 18.3 | 4.8 | 8 | 10 | 44 | 93 | Protein fate |
| 23 | gi|9656930 | VC2361 |  | formate acetyl transferase-related protein | 13.9 | 4.6 | 4 | 8 | 33 | 64 | Unknown function |
| 24 | gi|9655446 | VC0985 | *htpG* | heat shock protein HtpG | 72.2 | 5.1 | 24 | 21 | 53 | 178 | Protein fate |
| 25 | gi|9656400 | VC1869 | *pflA* | pyruvate formate-lyase 1 activating enzyme | 28.1 | 5.5 | 12 | 20 | 38 | 133 | Energy metabolism |
| 26 | gi|9654692 | VC0281 | *cadA* | lysine decarboxylase, inducible | 82.9 | 5.4 | 16 | 11 | 59 | 110 | Energy metabolism |
| 27 | gi|9655641 | VC1166 | *aspS* | aspartyl-tRNA synthetase | 65.8 | 5 | 19 | 10 | 66 | 205 | Protein synthesis |
| 28 | gi|9654410 | VC0018 | *ibpA* | 16 kDa heat shock protein A | 16.8 | 5.1 | 13 | 8 | 62 | 146 | Protein fate |
| 29 | gi|9656542 | VC2000 | *gapA-1* | glyceraldehyde 3-phosphate dehydrogenase | 35.3 | 6.1 | 8 | 0 | 100 | 120 | Energy metabolism |
| 30 | gi|9655600 | VC1128 | *trmU* | tRNA (5-methylaminomethyl-2-thiouridylate)-methyltransferase | 41.8 | 5.3 | 15 | 15 | 50 | 156 | Transcription |
| 31 | gi|9658435 | VCA0993 | *nemA* | N-ethylmaleimide reductase | 40.6 | 5.4 | 19 | 33 | 37 | 139 | Central intermediary metabolism |
| 32 | gi|9655567 | VC1098 | *ackA-1* | acetate kinase | 42.8 | 5.8 | 10 | 24 | 29 | 77 | Energy metabolism |
| 33 | gi|9655193 | VC0748 |  | aminotransferase NifS, class V | 44.8 | 6 | 12 | 13 | 48 | 124 | Central intermediary metabolism |
| Protein spots more abundant of N16961 cultured in sorbitol fermentation medium compared to fructose medium (SN/FN comparison) | | | | | | | | | | | |
| 35 | gi|9658166 | VCA0744 | *gplK* | glycerol kinase | 55.7 | 5.4 | 23 | 18 | 56 | 177 | Energy metabolism |
| 36 | gi|9657367 | VC2766 | *atpA* | ATP synthase F1, alpha subunit | 56.8 | 5.4 | 19 | 11 | 63 | 153 | Energy metabolism |
| 37 | gi|9657551 | VCA0161 | *tnaA* | tryptophanase | 52.9 | 6 | 12 | 7 | 63 | 116 | Energy metabolism |
| 38 | gi|9655788 | VC1300 | *sdaA-1* | L-serine dehydratase 1 | 49.3 | 6.1 | 19 | 17 | 53 | 131 | Energy metabolism |
| 39 | gi|9656634 | VC2086 | *sucB* | 2-oxoglutarate dehydrogenase, E2 component, dihydrolipoamide succinyltransferase | 44.1 | 5.5 | 10 | 17 | 37 | 55 | Energy metabolism |
| 40 | gi|9657923 | VCA0518 | *fruB* | PTS system, fructose-specific IIA/FPR component | 42.6 | 6 | 15 | 9 | 63 | 159 | Transport and binding proteins |
| 41 | gi|9654653 | VC0244 | *rfbE* | perosamine synthase | 41.1 | 6 | 17 | 5 | 77 | 197 | Cell envelope |
| 42 | gi|9658105 | VCA0687 |  | iron(III) ABC transporter, ATP-binding protein | 39.6 | 5.9 | 10 | 7 | 59 | 137 | Transport and binding proteins |
| 43 | gi|9657128 | VC2544 | *fbp* | fructose-1,6-bisphosphatase | 37.3 | 6.2 | 15 | 8 | 65 | 178 | Energy metabolism |
| 44 | gi|9656633 | VC2085 | *sucC* | succinyl-CoA synthase, beta subunit | 41.4 | 5.2 | 14 | 7 | 67 | 186 | Energy metabolism |
| 45 | gi|9657523 | VCA0136 | *glpQ* | glycerophosphoryl diester phosphodiesterase | 42.1 | 4.7 | 18 | 5 | 78 | 229 | Energy metabolism |
| 46 | gi|9654855 | VC0432 | *mdh* | malate dehydrogenase | 37 | 7.4 | 13 | 7 | 65 | 208 | Energy metabolism |
| 47 | gi|9658103 | VCA0685 |  | iron(III) ABC transporter, periplasmic iron-compound-binding protein | 37.7 | 6 | 15 | 11 | 58 | 175 | Transport and binding proteins |
| 48 | gi|9657922 | VCA0517 | *fruK* | 1-phosphofructokinase | 34.2 | 5.1 | 11 | 7 | 61 | 146 | Energy metabolism |
| 49 | gi|9656632 | VC2084 | *sucD* | succinyl-CoA synthase, alpha subunit | 29.9 | 6 | 11 | 6 | 65 | 153 | Energy metabolism |
| 50 | gi|9655912 | VC1415 | *hcp-1* | hcp protein | 19.1 | 5.2 | 6 | 7 | 46 | 77 | Unknown function |
| 51 | gi|9658051 | VCA0637 |  | oxygen-insensitive NAD(P)H nitroreductase | 24.1 | 5.1 | 8 | 4 | 67 | 120 | Central intermediary metabolism |
| 52 | gi|9658428 | VCA0987 | *ppsA* | phosphoenolpyruvate synthase | 88.5 | 4.7 | 23 | 9 | 72 | 228 | Energy metabolism |
| 53 | gi|9656637 | VC2088 | *sdhB* | succinate dehydrogenase, iron-sulfur protein | 26.4 | 5.4 | 10 | 6 | 63 | 141 | Energy metabolism |
| 54 | gi|9655459 | VC0997 | *glnS* | glutaminyl-tRNA synthetase | 64.1 | 5.2 | 14 | 14 | 50 | 132 | Protein synthesis |
| 55 | gi|9656635 | VC2087 | *sucA* | 2-oxoglutarate dehydrogenase, E1 component | 105.3 | 6.1 | 23 | 8 | 74 | 199 | Energy metabolism |
| 56 | gi|9658168 | VCA0748 | *glpB* | anaerobic glycerol-3-phosphate dehydrogenase, subunit A | 48 | 7.8 | 19 | 17 | 53 | 170 | Energy metabolism |
| 57 | gi|9657613 | VCA0220 | *hlyA* | haemolysin | 82 | 5.1 | 21 | 12 | 64 | 219 | Cellular processes |
| 58 | gi|9656638 | VC2089 | *sdhA* | succinate dehydrogenase | 64.5 | 6.5 | 27 | 7 | 79 | 263 | Energy metabolism |
| 59 | gi|9655682 | VC1203 | *hutU* | urocanate hydratase | 61.8 | 6.3 | 17 | 25 | 40 | 112 | Energy metabolism |
| 60 | gi|9657336 | VC2738 | *pckA* | phosphoenolpyruvate carboxykinase | 59.8 | 5.7 | 28 | 12 | 70 | 289 | Energy metabolism |
| Protein spots more abundant of N16961 cultured in fructose fermentation medium compared to fructose medium (SN/FN comparison) | | | | | | | | | | | |
| 62 | gi|9654902 | VC0474 | *irgB* | iron-regulated outer membrane virulence protein, TonB receptor family | 34.1 | 7.2 | 17 | 8 | 68 | 193 | Regulatory functions |
| 63 | gi|9655566 | VC1097 | *pta* | phosphate acetyltransferase | 76.9 | 5.1 | 28 | 7 | 80 | 281 | Energy metabolism |
| 64 | gi|9655446 | VC0985 | *htpG* | heat shock protein HtpG | 72.2 | 5.1 | 17 | 5 | 77 | 193 | Protein fate |
| 65 | gi|9657144 | VC2559 | *cysN* | sulfate adenylate transferase, subunit 1 | 52.9 | 5.6 | 14 | 9 | 61 | 147 | Central intermediary metabolism |
| 66 | gi|9657367 | VC2766 | *atpA* | ATP synthase F1, alpha subunit | 56.8 | 5.4 | 12 | 3 | 80 | 129 | Energy metabolism |
| 67 | gi|9654583 | VC0179 |  | hypothetical protein | 49.4 | 5.6 | 6 | 7 | 46 | 61 |  |
| 68 | gi|9655193 | VC0748 |  | aminotransferase NifS, class V | 44.8 | 6 | 10 | 3 | 77 | 126 | Central intermediary metabolism |
| 69 | gi|9655428 | VC0968 | *cysK* | cysteine synthase A | 34.2 | 6 | 13 | 3 | 81 | 221 | Amino acid biosynthesis |
| 70 | gi|9654905 | VC0478 | *fbaA* | fructose-bisphosphate aldolase, class II | 38.9 | 4.7 | 7 | 11 | 39 | 99 | Energy metabolism |
| 71 | gi|9654401 | VC0010 |  | amino acid ABC transporter, periplasmic amino acid-binding portion | 27.3 | 5 | 8 | 4 | 67 | 123 | Transport and binding proteins |
| 72 | gi|9655175 | VC0731 |  | antioxidant, AhpC/Tsa family | 22.9 | 5.3 | 9 | 4 | 69 | 124 | Cellular processes |
| 73 | gi|9658051 | VCA0637 |  | oxygen-insensitive NAD(P)H nitroreductase | 24.1 | 5.1 | 7 | 1 | 88 | 112 | Central intermediary metabolism |

#: The numbers are corresponding with the numbers marked on Fig. 3.

*: The scores greater than 48 were statistically significant (p<0.05).
